# The Study of Medicine, Surgery, and Anatomy; from the Creation of the World to the Commencement of the Nineteenth Century

**Published:** 1831-07

**Authors:** 


### BIBLIOGRAPHICAL NOTICE.

*The Study of Medicine, Surgery, and Anatomy; from the Creation of the World to the Commencement of the Nineteenth Century.* By WILLIAM HAMILTON, M.B. 2 vols. 8vo. Colburn and Bentley, London.

THIS is a work which the medical reader will peruse with interest and instruction, and the public in general with amusement. It cannot but be interesting to medical practitioners to trace their science from its early dawn to its present comparative state of maturity; to mark the progressive steps which have gradually led to the most splendid and useful discoveries in medicine, surgery, and anatomy; and to note the apparently trifling facts that have, at various epochs, laid the foundation for brilliant theories, and great practical improvements.

The compiler, like the lexicographer, is often looked upon as the mere "pioneer of literature," as one who requires only patient labour; but assuredly much judgment and solid learning are needed in the selection of matter, when such a huge, misshapen, and contradictory mass of materials is presented for his choice, as that with which the history of the science here treated of is encumbered. Dr. HAMILTON has been very successful in his management of the tedious and perplexing task he has undertaken; he has, in a concise form, given a very pleasing and instructive abstract of the history of medicine, surgery, and anatomy, from the earliest periods to the commencement of the 19th century.— The style in which the work is written is perspicuous and eloquent.

We have been much gratified by looking over the various anatomical preparations in wax which have been imported by Mr. Schloss. Among other elegant and very useful specimens, we may mention the human brain, after Reil and Vicq d'Azyr's method; the organ of hearing, after Dr. Bock's preparations; the human eye, larger than the natural size, displayed after the mode of Soemmering; and models relating to midwifery, after the original preparations and dissections of Dr. Kluge, of Berlin. Besides these preparations, models in wax of every part of the human body, both in a physiological and pathological point of view, may be had of the importer. Mr. Schloss has also lately published many splendid anatomical engravings, at a comparatively cheap price, which are well worth the attention of the profession. Weber's Anatomical Atlas, for example, has been submitted to the inspection of the first anatomists in this country, and has been highly approved, as a most important and instructive work both for students and practitioners.

---

